# Annular Lichenoid Dermatitis (of Youth)

**DOI:** 10.3390/dermatopathology9010004

**Published:** 2022-01-16

**Authors:** Giorgio Annessi, Emanuele Annessi

**Affiliations:** Istituto Dermopatico dell’Immacolata, Istituto di Ricovero e Cura a Carattere Scientifico (IRCCS), 00167 Rome, Italy

**Keywords:** lichenoid, annular, youth

## Abstract

About 20 years after its first description, Annular Lichenoid Dermatitis of Youth (ALDY) is recognized as a distinctive lichenoid dermatosis with specific clinical and histological features. The disease occurs mostly in young persons all over the world, runs a chronic course, and has an obscure etiopathogenesis. Clinically, lesions consist of persistent, asymptomatic erythematous macules and round-oval annular patches with a red-violaceous non-scaling border and central hypopigmentation, mostly localized on the groin and flanks. Histology shows a peculiar lichenoid dermatitis characterized by irregular epidermal hyperplasia with an alternation of thinned and quadrangular rete ridges and a dense band-like lichenoid infiltrate of lymphocytes in the papillary dermis. Typically, there is infiltration of lymphocytes into the lower epidermal layers with massive necrosis/apoptosis of keratinocytes, which is limited to the tips of rete ridges. Dermal lymphocytes are usually CD3^+^, CD4^+^, while most of the intraepidermal T cells are CD8^+^. Analysis of TCR-γ-chain gene rearrangement displayed polyclonality in all cases examined. Differential diagnosis mainly includes morphea, mycosis fungoides, annular erythemas and inflammatory lesions of vitiligo. Topical corticosteroids and topical tacrolimus represent the most effective drugs for ALDY treatment.

## 1. Introduction

In 2003, Annessi and colleagues described 23 Italian children and adolescents with red-violaceous annular patches that were mostly localized on the groin and flanks and showed a peculiar histologic lichenoid dermatitis with massive necrosis/apoptosis of the keratinocytes at the tips of the rete ridges. On the basis of these clinical, histological and epidemiological features, they proposed naming the disease Annular Lichenoid Dermatitis of Youth (ALDY) [1]. Since the original description, many other cases have been described worldwide, and ALDY is now considered to be a new clinicopathological entity that is mostly distinguished from annular erythemas, morphea and mycosis fungoides [2,3,4,5,6,7,8,9,10,11,12,13,14,15,16,17,18,19,20,21].

## 2. Epidemiology

ALDY occurs mainly in young persons (mean age 14.7 years, median age 10.5 years), although it has also been described in adults (range 2–79 years). The disease affects slightly more males than females (1.6:1). Although patients are mostly from the Mediterranean area, cases from central Europe, the United States [10,11], Japan [8], India [14] and Korea have also been reported [1,2,3,4,5,6,7,9,12].

ALDY patients were typically in good general health, without a remarkable medical history, except for a few patients who had atopic dermatitis, asthma, allergic rhinitis and celiac disease [1,6]. No association with drugs, tick bites, autoimmune disease or neoplasm has been found, and no seasonal clustering of the disease has been reported [1,2,3,4,5,6,7,8,9,10,11,12,13,14,15,16,17,18,19,20,21]. Laboratory studies, including complete blood cell count, erythrocyte sedimentation rate, general chemistry panel, stool examination for ova and parasites, rheumatoid factor and antinuclear antibody test, have been negative or within normal limits. The anti-streptolysin titer was elevated in three cases [1]. ALDY was not typically associated with signs of infection, and in fact, serum antibodies against cytomegalovirus, Epstein–Barr virus, parvovirus B19, Coxsackie/Echovirus and respiratory syncytial virus were negative or within normal limits in all patients [1,2,3,4,5,6,7,8,9,10,11,12,13,14,15,16,17,18,19,20,21].

Although serologic testing for *Borrelia burgdorferi* was found to be negative in all cases, in 11/14 specimens from a series of Western Austrian patients, Borrelia microorganisms, either vital or degenerated, were detected in close proximity of collagen bundles [16].

Standard patch tests were performed in many cases, always with negative results [1,2]. In one case, ALDY occurred after anti-hepatitis B vaccination [5].

## 3. Clinical Features

**Figure 1:**
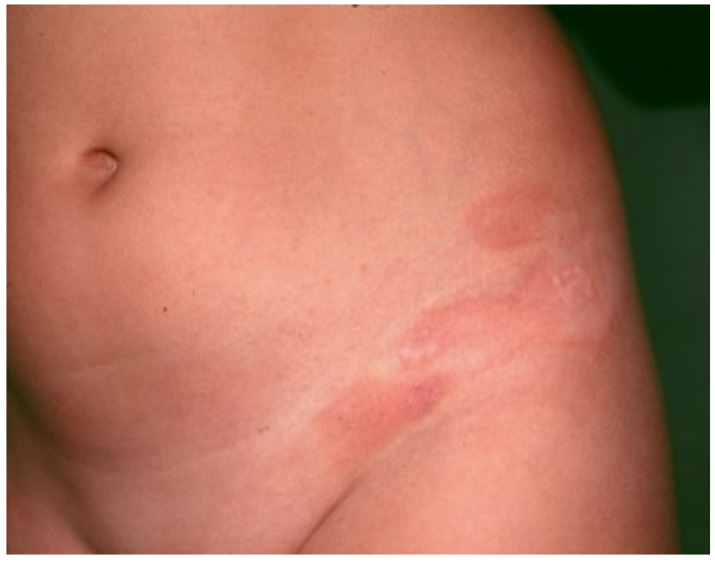
Early lesions consist of erythematous macules and vaguely annular patches with a slightly raised border and irregular zones of central clearing on the groin and flank.

**Figure 2:**
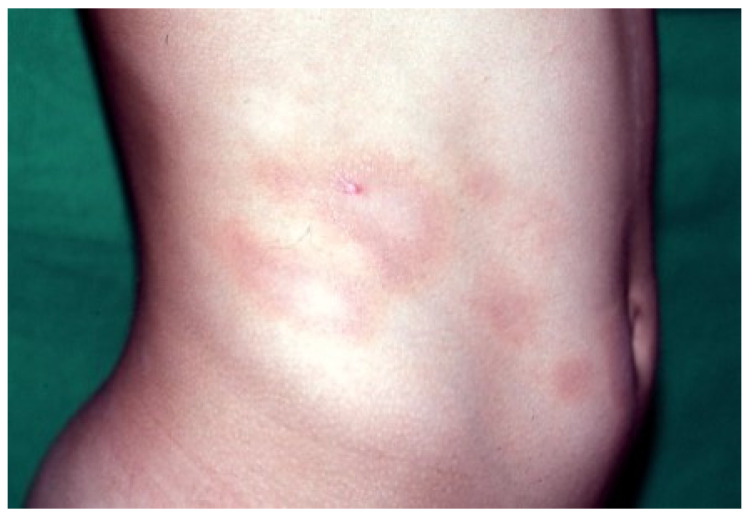
Fully developed lesions appear as annular patches with red-violaceous, non-scaling border and central areas of hypopigmentation on the flank and abdomen.

**Figure 3:**
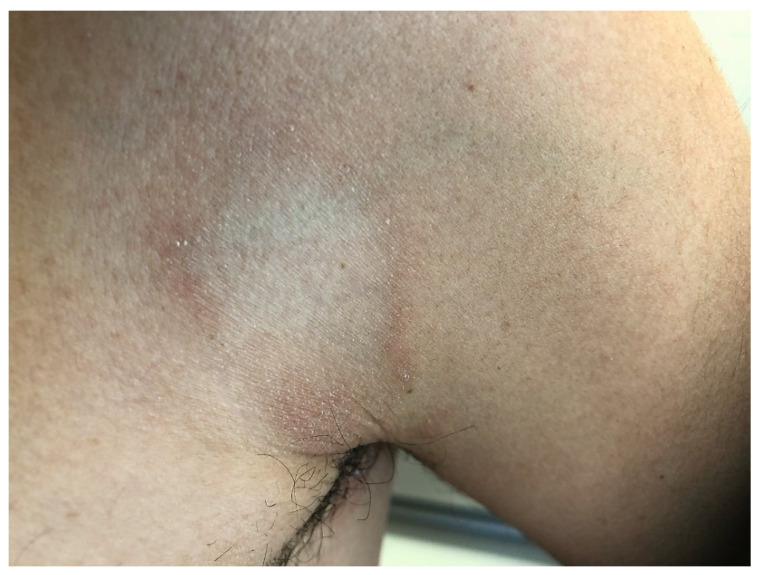
Late lesion is characterized by a complete ring of hyperpigmentation on the axilla.

ALDY lesions can have different morphologies according to the stage of the disease. Early lesions consist of asymptomatic, round-oval erythematous macules 1–5 cm in diameter with a relatively sharp border and smooth surface. Some of the macules may enlarge centrifugally, giving rise to vaguely annular patches with a slightly raised erythematous border and irregular areas of central clearing (Figure 1). Fully developed lesions appear as round-oval annular patches, 5–15 cm in diameter, with a red-violaceous or red-brownish non-scaling border delimiting a central zone of hypopigmentation (Figure 2). Rarely, pinpoint papules are seen at the periphery of annular patches. At times, annular patches either merge to form figurate lesions or dissolve into incomplete red-violaceous rings. In older lesions, the peripheral erythema tends to fade, leaving complete or incomplete rings of hyperpigmentation that surround whitish non-atrophic and non-indurated areas (Figure 3) [1,12,13,18].

Although the disease may occasionally occur with solitary lesions, most patients present with multiple annular patches that characteristically involve the trunk. In particular, sites of predilection appear to be the groin, flanks, peri-umbilical region and, less frequently, the axillary region and neck. Rare sites are the upper arm and calf. The condition is usually asymptomatic except for occasional mild pruritus during the development of new macules.

## 4. Histopathology

**Figure 4:**
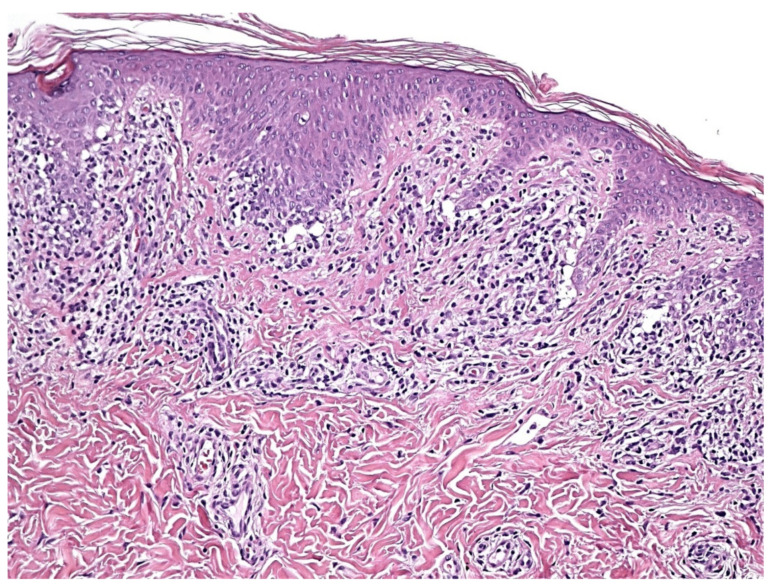
Histologically, early lesions show a lichenoid dermal infiltrate of lymphocytes at the tips of rete ridges, where vacuolar changes and some necrotic/apoptotic keratinocytes are seen at the dermo-epidermal junction. The horny layer has a basket-weave appearance, and the granular layer is normal.

**Figure 5:**
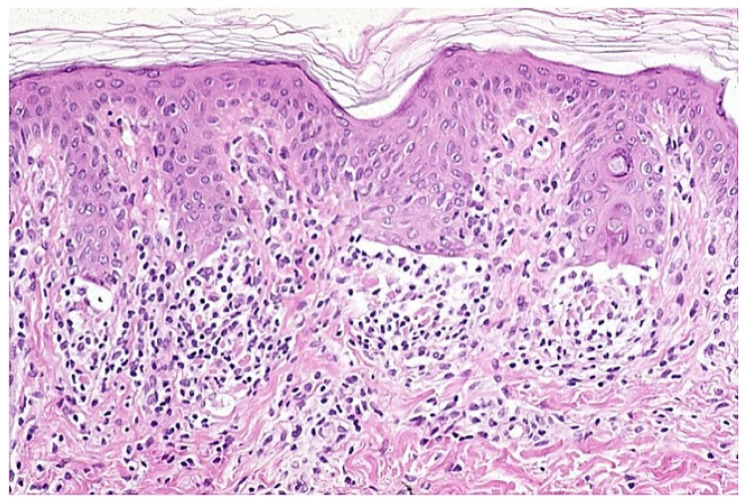
There is a dense lichenoid infiltrate of lymphocytes limited to the tips of rete ridges, which acquire a quadrangular shape as a consequence of massive necrosis/apoptosis of keratinocytes. Note that the epidermal supra-papillary plates are spared by the inflammatory infiltrate.

**Figure 6:**
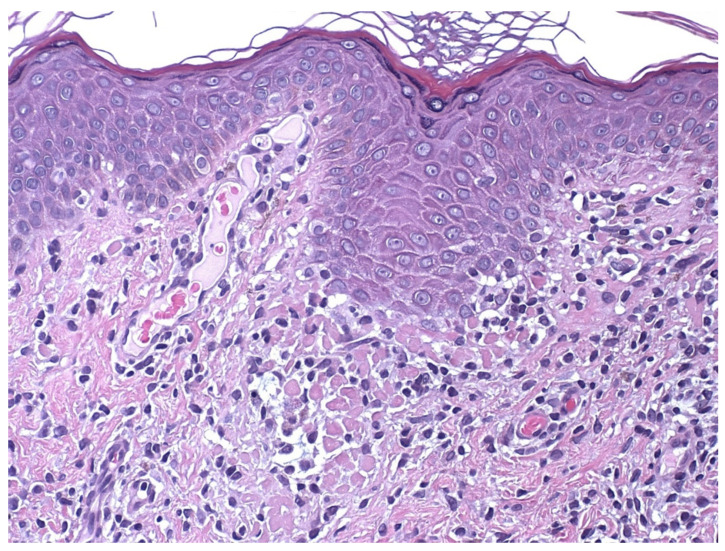
Massive necrosis/apoptosis of keratinocytes with “decapitation” of the base of an epidermal ridge.

**Figure 7:**
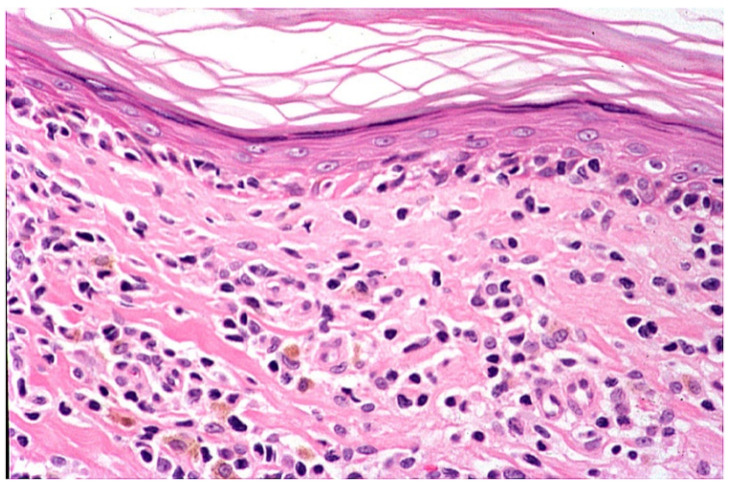
Late lesion shows a flattening of the epidermis, focal areas of vacuolar alteration and sparse necrotic keratinocytes at the dermo-epidermal junction. Numerous melanophages are seen in the papillary dermis.

Early erythematous macules show mildly elongated and thinned rete ridges. Unlike other lichenoid dermatoses, the horny layer has a basket-weave appearance, and the granular layer is of normal thickness. A vaguely band-like infiltrate of lymphocytes is present in the papillary dermis. Vacuolar alteration, together with occasional necrotic/apoptotic keratinocytes and obscuring of the dermo-epidermal junction by lymphocytes, is exclusively observed at the tips of rete ridges (Figure 4). Fully developed annular patches display distinctive irregular epidermal hyperplasia characterized by an alternation of thinned and quadrangular rete ridges. A dense band-like lichenoid infiltrate of lymphocytes is seen in the papillary dermis. Typically, the infiltration of small lymphocytes into the lower epidermal layers with massive necrosis/apoptosis of keratinocytes is limited to the tips of rete ridges while sparing the supra-papillary plates of the epidermis (Figure 5 and Figure 6). Hyperpigmented borders of late lesions show a flattening of the epidermis, focal areas of vacuolar alteration and sparse necrotic keratinocytes at the dermo-epidermal junction (Figure 7). Lymphocytic infiltrates, occasionally obscuring the dermo-epidermal junction, and several melanophages are present in the papillary dermis. Central hypopigmented areas of both fully developed and late annular patches display epidermal flattening and superficial perivascular infiltrates of lymphocytes, together with sparse melanophages [1,12,13,18].

## 5. Immunohistochemical and Molecular Analysis 

Immunohistochemical studies have revealed dermal and epidermal infiltrates consisting of CD3^+^, CD2^+^, CD5^+^ and CD7^+^ lymphocytes. Characteristically, dermal lymphocytes are CD4^+^, whereas most of the intraepidermal T cells are CD8^+^. No or few CD20^+^ cells and some CD34^+^, CD68^+^ or CD138^+^ cells may be observed within the infiltrate. CD1a staining shows an increased number of suprabasal intraepidermal Langerhans cells. In biopsy specimens from central hypopigmented areas, Melan-A antibody demonstrated a normal number of melanocytes in the basal layer of the epidermis, while Fontana–Masson staining showed a scant amount of melanin granules in basal keratinocytes. To date, the analysis of TCR-γ-chain gene rearrangement has revealed polyclonality in all cases examined. Moreover, polyclonality has been confirmed in skin samples from recurrent lesions in some patients [1,12,13,18].

## 6. Course

ALDY runs a chronic course with little tendency to extend to and involve previously unaffected areas. Single lesions usually persist for months or years, and the disease undergoes spontaneous resolution in only a few cases [1,12,13,18].

## 7. Therapy 

Topical corticosteroids have been shown to be the most effective drugs for ALDY treatment [1,21]. The use of potent corticosteroids ointments usually results in marked improvement or complete regression of annular patches with complete and homogeneous repigmentation of hypopigmented areas. The lesions, however, may recur after treatment discontinuation. In some cases, disease remission has also been obtained with topical tacrolimus [6,12,14] and pimecrolimus [15,18] ointment. Other treatments used include systemic cyclosporin [20], systemic corticosteroids, heliotherapy, phototherapy (UV-A), photochemotherapy (psoralen-UV-A) and systemic (doxycycline, macrolides and cephalosporins) and topical antibiotics (mupirocin and gentamicin) (Table 1).

## 8. Differential Diagnosis 

Differential diagnosis mainly includes morphea, mycoses fungoides and annular erythemas [1,12,13,18]. In contrast to ALDY, inflammatory plaques of morphea are typically indurated with an atrophic, shiny and hairless surface [22]. Moreover, late ALDY patches have complete or incomplete rings of hyperpigmentation, while pigmentary changes in morphea tend to have a random distribution within the plaques. Histologically, inflammatory lesions of morphea show superficial and deep perivascular and interstitial infiltrates of lymphocytes, histiocytes and plasma cells in combination with early sclerosis of the papillary and/or reticular dermis.

In some cases, early erythematous macules and annular lesions of ALDY may clinically resemble mycosis fungoides. Patches and plaques of mycosis fungoides, however, have a wrinkled surface partially covered by thin scales. Moreover, the presence of an erythematous or brownish border around areas of hypopigmentation differentiates ALDY annular patches from those of the hypopigmented type of mycosis fungoides. Histologically, ALDY may be distinguished from mycosis fungoides by an infiltration of lymphocytes within the epidermis restricted to the tips of rete ridges, the finding of entire rete ridges transformed into clusters of necrotic/apoptotic keratinocytes, the lack of alignment of lymphocytes in the basal layer of the epidermis along a wide front of the lesion and the absence of atypical lymphocytes, and eosinophils or plasma cells in the dermal infiltrate. In doubtful cases, analysis of the rearrangements of TCR genes may be an adjunctive tool helpful in distinguishing ALDY from mycoses fungoides [1,23].

Often, ALDY may resemble annular erythemas, such as erythema annulare centrifugum [24] and annular erythema of infancy [25]. However, the typical collarette of scale seen at the inner edge of the advancing border of erythema annulare centrifugun is never observed in ALDY, and lesions of annular erythema of infancy do not resolve with residual hypopigmentation and hyperpigmentation. Furthermore, no annular erythema shows the typical histopathologic lichenoid pattern seen in ALDY.

Finally, inflammatory lesions of vitiligo [26] consisting of depigmented patches surrounded by an erythematous and scaling border can be histologically differentiated from ALDY by the presence of a vaguely lichenoid dermal infiltrate that is not limited to the tips of the rete ridges and the lack of massive necrosis/apoptosis of keratinocytes.

## 9. Etiopathogenesis

Currently, the etiopathogenesis of ALDY is unknown (1–21), although immunohistochemistry findings suggest that the disease may be caused by a cytotoxic T-cell-mediated immune reaction, as is the case in other lichenoid skin reactions [27]. As mentioned above, only one study from Western Austria found the presence of Borrelia or Borrelia-like microorganisms in ALDY lesions [16]. According to the authors, this finding indicates that Borrelia might play a role in ALDY etiopathogenesis and that, at least in some cases, the disease might represent another form of a superficial stage of morphea besides lichen sclerosus et atrophicus. We disagree with these hypotheses for the following reasons: (a) The fact that systemic antibiotic treatment for Borrelia has proved to be ineffective [1,10,16] militates against the etiologic role of this microorganism in ALDY. (b) Clinically, the central areas of the more superficial form of morphea tend, over time, to become sclerotic, indurated and hairless. In contrast, annular patches of ALDY are never indurated and heal spontaneously or after treatment without sequelae. Lichen sclerosus et atrophicus is characterized by polygonal, white-ivory, shiny, slightly elevated interfollicular papules that, over time, often enlarge or coalesce into larger plaques. In a more advanced stage, telangiectasias or follicular plugging can be seen. None of these clinical features are observed in ALDY. (c) Histologically, the superficial form of morphea never presents with lichenoid changes. Early lesions of lichen sclerosus et atrophicus may indeed show focal lichenoid infiltrates of lymphocytes; however, they are always accompanied by initial fibrosclerosis of the papillary dermis [28]. By contrast, it must be emphasized that dermal fibrosis or sclerosis is never a histologic feature of any stage of ALDY [1]. (d) ALDY lesions positive for Borrelia were characterized by scaling, wrinkling and dermal atrophy;^16^ over time, fibrosis and telangiectasias were very prominent, resembling chronic radiation dermatitis. Since these clinical features have never been described before in ALDY, it is possible that at least some of the Austrian cases associated with Borrelia have been misinterpreted as ALDY.

## 10. Conclusions

In summary, about 20 years after its first description, ALDY appears to be a distinctive and unusual lichenoid dermatosis with specific clinical and histological features. The disease affects mostly young individuals all over the world, runs a chronic course, and has an obscure etiopathogenesis.

## Figures and Tables

**Table 1:** ALDY treatment review.

Reference (First Author)	Number of Cases	Therapy	Remission
Annessi	23	Tcs (17)	Complete
		Scs (1)	Complete
		A (4)	No remission
		SE (2)	Partial
		Ph (2)	Complete
De la Torre	1	Tcs	Complete
Durdu	1	Tcs	Complete
Kleikamp	1	Tcs and T	Complete
Cesinaro	3	Tcs (2)	Complete
		T (1)	
Huh	1	Tcs	Partial
Fabroni	1	Tcs	Complete
Leger	1	Tcs and Scs	Complete
Kazlouskaya	1	Tcs	Complete
Di Mercurio	6	Tcs (6)	Complete
		T (2)	Complete
Osorio	2	-	Spontaneous remission
Ulkumen	1	T	Complete
Malakhowski	1	Tcs and Pim	Complete
Wilk	12	Tcs (4)	Complete
		A (2)	No remission
Cesinaro	1	Tcs	Complete
Debois	1	Tcs and Pim	Complete
Sans	1	Tcs	Complete
Mahmoudi	3	Tcs and T (3)	complete
Stojkovic-Filipovic	1	Cy	Complete

Tcs: topical corticosteroids; Scs: systemic corticosteroids; A: antibiotics; SE: sun exposure; Ph: phototherapy; T: tacrolimus ointment; Pim: pimecrolimus ointment; Cy: cyclosporine.

## Data Availability

Not applicable.
